# The association of psychological distress and economic and health worries with tobacco smoking behavior during the COVID-19 pandemic: a two-year longitudinal cohort study

**DOI:** 10.1186/s12889-024-17943-x

**Published:** 2024-02-05

**Authors:** Silvia Eiken Alpers, Karl Trygve Druckrey-Fiskaaen, Tesfaye Madebo, Jørn Henrik Vold, Ståle Pallesen, Jens Christoffer Skogen, Linn-Heidi Lunde, Silje Mæland, Lars Thore Fadnes

**Affiliations:** 1 Department of Addiction Medicine, Haukeland University Hospital, Bergen, Norway; 2 Department of Psychosocial Science, Faculty of Psychology, University of Bergen, Bergen, Norway; 3 Bergen Addiction Research, Department of Addiction Medicine, Haukeland University Hospital, Bergen, Norway; 4 Department of Global Public Health and Primary Care, Faculty of Medicine, University of Bergen, Bergen, Norway; 5 Department of Respiratory Medicine, Stavanger University Hospital, Stavanger, Norway; 6 Department of Clinical Sciences, University of Bergen, Bergen, Norway; 7 Division of Psychiatry, Haukeland University Hospital, Bergen, Norway; 8 Department of Health Promotion, Norwegian Institute of Public Health, Bergen, Norway; 9 Alcohol & Drug Research Western Norway, Stavanger University Hospital, Stavanger, Norway; 10 Centre for Evaluation of Public Health Measures, Norwegian Institute of Public Health, Oslo, Norway; 11 Department of Clinical Psychology, Faculty of Psychology, University of Bergen, Bergen, Norway

**Keywords:** COVID-19, Smoking, Tobacco, Psychological distress, Worries, Risk factors, Pandemic

## Abstract

**Background:**

The COVID-19 pandemic and other life events may trigger worries and psychological distress. These impacts may lead to unhealthy behaviors, such as tobacco smoking, but the degree of such associations is unclear. The current three-wave longitudinal study examines changes in tobacco smoking in Norway between 2020 and 2022 and their associations with psychological distress as well as health- and economy-related worries.

**Methods:**

Data were collected in April 2020 (baseline), January 2021, and January 2022 in Bergen, Norway, from an online longitudinal population-based survey. Smoking tobacco (the outcome variable) was dichotomized based on the responses to the question of whether participants smoked cigarettes or not. Tobacco smoking and its associations with psychological distress were assessed among 24,914 participants (response rate 36%) in a mixed model regression presented with coefficients and 95% confidence intervals (CI), adjusting for COVID-19-related worries, home office/study, occupational situation, age, gender, education, having children below 18 years living at home, living alone, and alcohol consumption.

**Results:**

A total of 10% of the study sample were current smokers at baseline. At baseline, smoking tobacco was associated with high levels of psychological distress (absolute difference 13%, 95% CI 10%; 15%), advanced age (50−59 years: 11%, CI 10%; 13%), and hazardous alcohol use (4%, CI 3%; 5%) compared to their counterparts. Higher education (-5%, CI -6%; -4%), working from home (-4%, CI -5%; -4%), and higher physical activity levels (-4%, CI -5%; -3%) were associated with non-smoking. The prevalence of smoking among individuals experiencing severe psychological distress decreased slightly over time (-2% per year, CI -3%; -1%).

**Conclusions:**

Smoking was associated with severe psychological distress, advanced age, and hazardous alcohol use at baseline; non-smoking was associated with high education, working from home, and high physical activity. Nevertheless, the smoking rate among individuals experiencing severe psychological distress slightly decreased over the course of the COVID-19 pandemic.

**Supplementary Information:**

The online version contains supplementary material available at 10.1186/s12889-024-17943-x.

## Background

Tobacco smoking has long been recognized as a significant public health concern, contributing to an estimated 7.69 million deaths and 200 million disability-adjusted life years in 2019 [1]. Several studies have investigated the relationship between tobacco smoking and diverse factors, including psychological distress, educational attainment, employment status, and various social and lifestyle aspects. One specific study showed a consistently higher prevalence of psychological distress among younger smokers with lower levels of formal education and smokers with lower incomes, in contrast to their higher-income counterparts [2]. Another study reported an increase in serious psychological distress among smokers over time [3]. Other studies have shown that quitting smoking has been linked to positive improvement in mental health [4], physical activity (PA) levels [5], and patterns of alcohol consumption [6]. Growing evidence indicates that cigarette smoking plays a major role in increasing the risk of adverse COVID-19 outcomes [7]. The COVID-19 pandemic was a global shock, causing severe medical, economic, and personal distress, and there is evidence that being a smoker is associated with lower psychological distress tolerance [8]. Accordingly, it might be expected that tobacco smoking would increase during the COVID-19 pandemic. A systematic review, however, found that overall tobacco consumption decreased during the COVID-19 pandemic, although this effect varied among the included countries. Iceland and some states in the United States reported increased smoking prevalence [9], whereas countries such as China, Pakistan, the United Kingdom, and Spain reported reductions in the percentage of smokers. These countries or states had the most stringent COVID-19 policies. Reduced social contact and fear of more serious COVID-19 outcomes were postulated as reasons for the decrease in tobacco consumption [9]. In regions with increased tobacco consumption, the rise was attributed to higher levels of psychological distress and boredom [9].

Studying tobacco usage in the context of the COVID-19 pandemic holds important implications for public health, extending its relevance beyond the immediate crisis. The insights gained not only assist current public health responses but also lay the foundation for more effective future strategies. These strategies aim to enhance respiratory health, alleviate health disparities—especially by customizing approaches for high-risk groups—and understanding the dynamics of health-related behaviors. Mental health challenges persist beyond the pandemic. Recognizing the link between mental health and tobacco use informs strategies for supporting individuals in managing stress and anxiety without resorting to harmful behaviors. Additionally, such studies enable better comprehensive public health planning and preparedness for potential future crises involving similar infectious diseases.

In Norway, the most stringent COVID-19 measures were implemented on March 12, 2020. These were the strongest restrictions in Norway since World War II, although they did not amount to a complete lockdown. To better prepare for future crises, it is imperative to raise awareness about smoking behaviors and prioritize targeted interventions, including smoking cessation programs, mental health support, and tailored approaches for high-risk groups.

The aim of this study was to assess changes in tobacco smoking across three longitudinal waves during the COVID-19 pandemic, and examine whether psychological distress, health and economic worries, social and demographic factors, PA levels, alcohol consumption, job uncertainty/situation, and education were associated with tobacco smoking.

## Materials and methods

### Data source

**Fig. 1:**
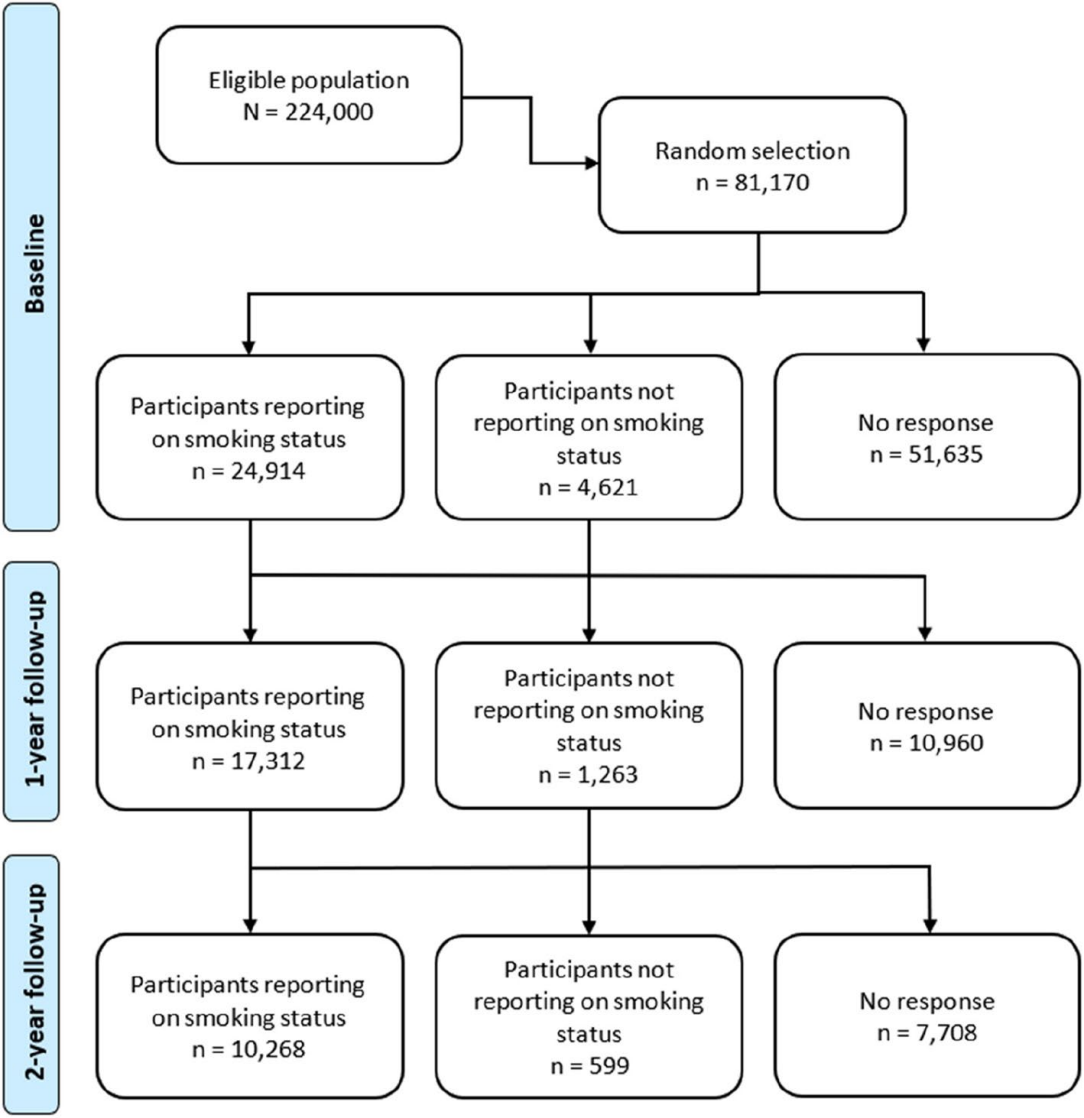
Flow chart of the study design and cohort overview. Flow chart of the longitudinal study design, including wave 1, which consisted of a two-week baseline assessment (T0), wave 2 after nine months, covering four weeks of data collection (T1), and wave 3 after an additional 12 months, covering four weeks of data collection (T2)

In April 2020, an invitation to participate in the "Bergen in Change" (BiE-study) was extended to a representative sample of 81,170 people from among the 224,000 adult residents of Bergen, Western Norway. The BiE-study aimed to examine the potential effects of the lockdown on daily life, health, and health behaviors during the COVID-19 pandemic [10]. The sample was selected randomly and mirrored the Norwegian speaking general population concerning age and gender. Those invited to participate were drawn from the National Population Registry of Norway and the common contact register^1^. In total, 29,535 people (response rate 36%) agreed to take part in the first wave (T0) of the present study (Fig. 1).

### Data collection

We administered a set of electronic questionnaires in Norwegian using email and short text messages (SMS) via the online data collection tool SurveyXact (surveyxact.no). The questionnaire collected demographic information and posed questions related to various aspects of life and health concerning the COVID-19 pandemic and lockdown. The initial data collection (T0/baseline) occurred from April 15 to April 30, 2020. A month before T0, several COVID-19-related restrictions came into effect, including social distancing, closure of educational, cultural, and training/sport/gym facilities, requirements for remote work, and the introduction of quarantine protocols.

All participants from T0 were invited to participate in the survey at T1, which was conducted between December 2020 and January 2021. By this time, restrictions had been eased slightly. Schools had reopened, organized sports activities were gradually resumed, and restaurants and cafes operated with limited capacity. Nevertheless, social distancing and advice to avoid public transport were still in place [11]. In January 2021, in response to a new wave of the virus, restrictions were reintroduced, coinciding with the latter part of T1. At T1, 18,575 people took part in the survey (a follow-up rate of 63% from T0), with a median time interval of nine months from T0.

The third wave of data collection (T2) took place between December 2021 and January 2022, aligning with the end of most pandemic-related measures. Many restrictions imposed in April 2020 were then lifted. However, people were still encouraged to practice social distancing and wear face masks in shops and public institutions while the focus on maintaining a one-meter distance was reduced. The median time interval between T1 and T2 was 12 months. In total, 10,867 respondents participated in the third wave (a follow-up rate of 37% from T0). The older age groups were substantially less likely to be lost to follow-up than the younger adults, and similarly those with higher education were less likely to drop off compared to those with lower education, while men were slightly more likely to be lost to follow-up than women (see Additional Table 1).

**Table 1:** Background characteristics of participants at baseline and follow-up

**Total**	**Baseline** ***n* (%)**	**1-year follow-up** ***n* (%)**	**2-year follow-up** ***n* (%)**
*n*	24,914 (100%)	17,312 (100%)	10,268 (100%)
Gender (women)	14,079 (57%)	9761 (56%)	5896 (57%)
Primary school	1856 (8%)	1076 (7%)	599 (6%)
High school	6978 (28%)	4403 (27%)	2542 (26%)
University ≤ 3 years	5948 (24%)	3954 (24%)	2333 (24%)
University > 3 years	9968 (40%)	6883 (42%)	4325 (44%)
Adjusted income (EUR) ^a^			
0–25,000	3005(14%)	1592 (11%)	876 (10%)
25,000–50,000	9695 (44%)	6359 (43%)	3804 (43%)
>50,000	9485 (43%)	6797 (46%)	4205 (47%)
Persons in household			
1	5049 (21%)	3514 (22%)	2188 (23%)
2	7864 (33%)	5442 (34%)	3384 (35%)
3–4	8436 (35%)	5271 (33%)	3079 (32%)
5+	2809 (12%)	1710 (11%)	935 (10%)
Employment	16,769 (67%)	10,955 (63%)	6478 (63%)
Student/school	1958 (8%)	841 (5%)	456 (4%)
Placed in quarantine	4035 (16%)	2808 (16%)	1629 (16%)
Temporarily laid-off	1845 (8%)	1078 (6%)	581 (6%)
Home office/study	12,229 (49%)	8286 (48%)	4841 (47%)
COVID-19 symptoms	1531 (6%)	987 (6%)	595 (6%)
Worries	12,634 (51%)	7979 (46%)	4613 (45%)
Worries related to economy	4014 (16%)	2304 (13%)	1275 (12%)
Health-related worries	10,979 (44%)	6995 (40%)	4052 (39%)
Psychological distress	4872 (20%)	2896 (17%)	1672 (16%)
Smoking	2402 (10%)	1489 (9%)	796 (8%)
PA level			
Low	5645 (25%)	3614 (24%)	2176 (24%)
Moderate	9714 (44%)	6709 (45%)	4078 (45%)
High	6895 (31%)	4653 (31%)	2777 (31%)
Hazardous drinking	13,117 (53%)	8742 (54%)	5212 (53%)

^a^The adjusted income is the household income divided by the personal index. The personal index is calculated as 1 for the first adult, 0.7 per other adult household member, and 0.5 per child. The adjusted income was converted to Euros

Tables for background characteristics of participants per age group at baseline, 1-year follow-up, and 2-year follow-up separately are available in the Additional Tables 2-4

### Measures

The main outcome variable in the present study was self-reported tobacco smoking. The questions about smoking behavior differed across the three questionnaires (see Appendix). We had information on both smoking and snus use from T0 (in 2020). However, in the follow-up questionnaires (2021 and 2022), we chose to prioritize tracking changes in tobacco smoking over time. As a result, we do not have information on potential changes in the use of snus. In 2020, participants were asked if they currently smoked cigarettes or used snus and whether they had used more or less snus in the past month compared to the previous period. Those who answered “yes” to the first question and “I have not used snus in recent months” to the second question were categorized as smokers, while those who answered “no” to the first question were categorized as non-smokers. In 2021 and 2022, participants were simply asked, “Do you currently smoke cigarettes?” with “yes” or “no” responses, thus defining smokers vs. non-smokers. At T1 and T2, we also asked about the number of cigarettes the participants usually smoked per day.

Exposure variables were gender, age, education, children <18 years at home, living alone, alcohol consumption, psychological distress, PA level, COVID-19-related worries, and perceived lockdown consequences of the pandemic measures. The variables “being temporarily laid-off” and “having home office/studying from home” were designed as dichotomous variables and formulated as true/false statements. The questionnaire included two questions on economic worries related to fear of losing one’s job or worsening one’s economic situation. The response alternatives were “strongly agree”, “agree”, and “disagree”. Categorization of “economic worries” was based on answering at least one of the two questions with “strongly agree”. The “health worries” variable reflected how COVID-19 may affect one’s or others’ health and being anxious or worried that the infection would affect themselves, close family/loved ones, or elderly members of the family (with response alternatives strongly agree/agree/disagree). We defined health worries as strongly agreeing with at least one of the questions. For more detailed information see Alpers et al. [12].

In the questionnaire, education was categorized into four groups: primary school, high school, university ≤ 3 years, and university > 3 years. For use in the regression model, education was divided into lower and higher education. Higher education was classified as completing more than three years of studies at university or college.

We assessed psychological distress using the Norwegian-validated translation of the ten-item version of the Hopkins Symptom Checklist (SCL-10) [13]. Participants rated the frequency of experiencing symptoms of anxiety and depression over the past seven days on a 4-point Likert scale, ranging from 1 (not at all) to 4 (extremely). The mean score of the items served as an indicator of psychological distress. We created a dichotomous variable to identify participants with a mean score above 1.85 in the full-range score (1-4), which is a recognized cut-off value for predicting severe psychological distress [13]. This variable was used in the description of background characteristics of the participants. This mean score was then transformed into a continuous scale ranging from 0 to 1, which are also presented on a percentage scale, with 0 indicating no psychological distress and 1 indicating severe psychological distress. This variable was used in the regression model.

To assess PA levels, we employed the short form of the International Physical Activity Questionnaire (IPAQ-SF) [14]. The IPAQ-SF questions allowed us to measure the total weekly energy expenditure related to PA among the participants. This metric encompassed the cumulative time spent walking, moderate-intensity PAs, and vigorous-intensity PAs, quantified in metabolic equivalent task minutes per week (METs/week). In accordance with the IPAQ-SF scoring guidelines (http://www.ipaq.ki.se), high PA was defined as engaging in more than one hour of moderate-intensity activity beyond basal activity or over 30 minutes of vigorous-intensity activity above basal levels each day. Moderate activity referred to at least 30 minutes of moderate-intensity activity on most days of the week, while low activity encompassed individuals not meeting these criteria. Consequently, we categorized participants into three PA classes: low, moderate, and high.

Alcohol consumption was assessed by the Alcohol Use Disorders Identification Test Consumption (AUDIT-C), which consists of the first three questions of the full AUDIT [15, 16] (Appendix). Each question is scored using a 5-point scale ranging from 0 to 4; thus, the composite score of the AUDIT-C ranges from 0 to 12. In the present study, we determined hazardous drinking by using an AUDIT-C cut-off score of three for women and four for men. The cut-offs were set in accordance with established criteria [17, 18].

### Statistical analyses

We conducted all descriptive and regression model analyses using Stata/SE 17.0 (StataCorp, College Station, TX, USA). For a graphical presentation of changes in categories of tobacco use over time, we utilized Sankeymatic (sankeymatic.com). The threshold for statistical significance was set at *p*<0.05 for all analyses. In all our analyses, we defined time as the number of years from baseline. The response rate varied, with lower rates among younger individuals, men, and individuals with lower educational levels, and conversely being higher among older age groups, women, and those with higher educational levels. To address this imbalance, we stratified analyses by age group and employed inverse probability weights based on age and gender (education was not available for non-respondents and thus not included), aligning the sample more with the background population distribution. These weights were calculated using binomial regression models, with mean weights of 1.0 and a standard deviation of 0.25, ensuring a more representative representation in the final estimates. Weighted estimates for the exposure variables are presented along with their corresponding 95% confidence intervals (CI). We used chi-square tests to investigate statistically significant differences between groups of categorical variables.

To examine the association of exposure variables with tobacco smoking at baseline and the extent to which they were linked to changes in smoking over time, we used linear mixed model analyses. The outcome variable was dichotomized based on the responses to the question of whether participants smoked cigarettes or not (*n* = 24,914; 84% of the total sample) (yes (1) = smoker, no (0) = non-smoker). Throughout the analyses, the exposure variables remained constant at their baseline levels when predicting the levels and changes in the outcome variables. To explore whether exposure variables predicted changes in outcomes, we introduced interactions between these variables and time into the model. We used maximum likelihood estimation and included all available responses to the outcome variables in the analyses.

### Ethics approval and consent to participate

Participants in the present study provided informed consent before answering the questionnaires. They were assured of the confidentiality of their responses and their right to withdraw from participation at any time. The project received approval from the Regional Committee for Medical and Health Research Ethics, Health Region West, with the ethics registration code 2020/131560. Additionally, the study was conducted under guidance from data protection officials at the University of Bergen.

## Results

### Study sample

At baseline (T0), the median age of the participants was 50 years (interquartile range (IQR) 36−63), 57% were women, 40% had more than three years of university or college education, 94% were Norwegian citizens, 87% had a person-adjusted household income above 25,000 euros (EUR 1 ≈ 10 NOK), 67% were employed/worked, and 8% were students (Table 1). Two-thirds lived with 1−3 other people. The distribution of the age categories was similar across all three measurement points.

### Tobacco smoking

At T0, 10% of the participants reported tobacco smoking, which decreased to approximately 8% at T1 and T2.

**Fig. 2:**
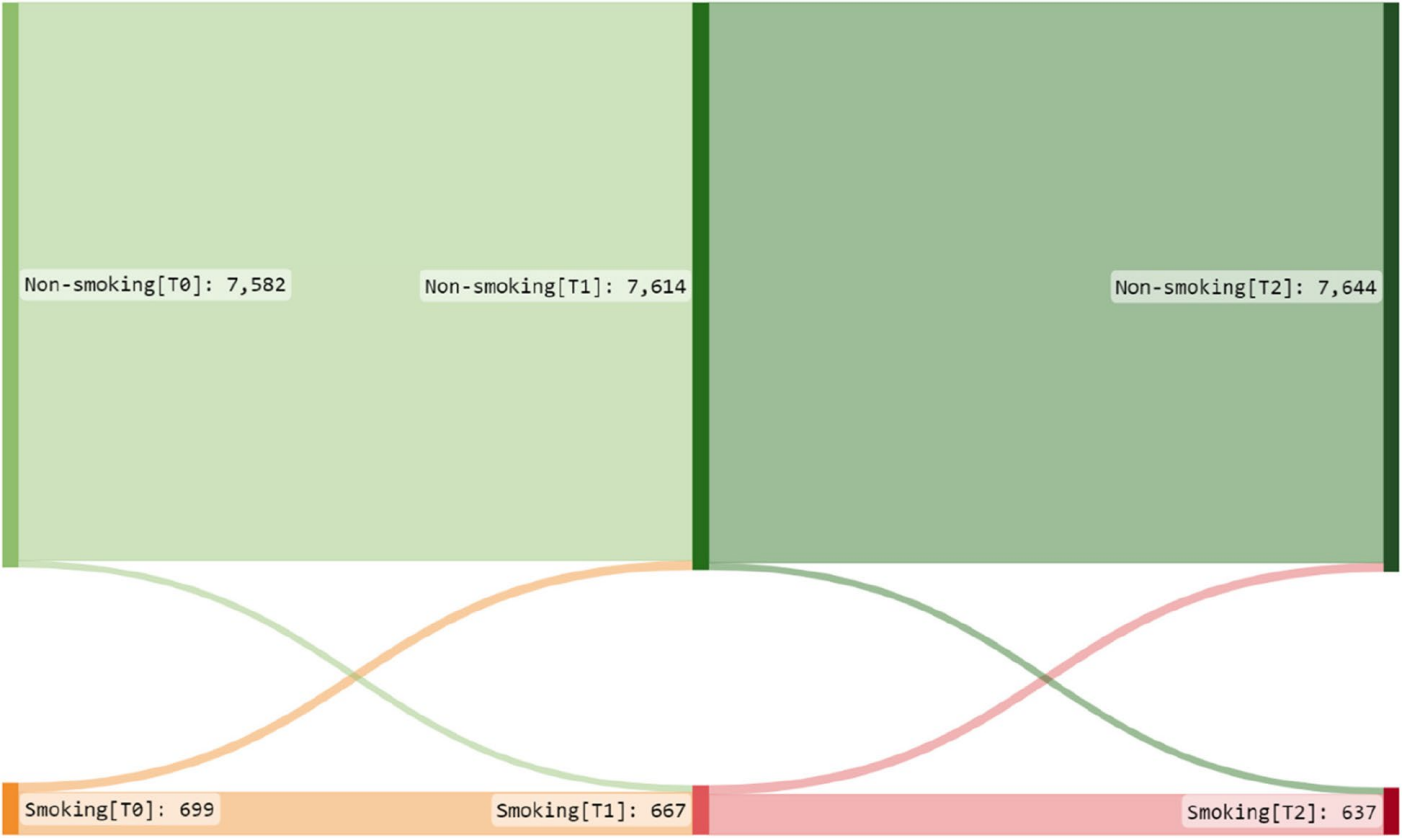
Sankey diagram of change in smoking behavior. The diagram shows smoking behavior broken down into two categories (smoking and non-smoking) at three time points (T0, T1, and T2). The paths show the proportion of individuals changing or not changing smoking behavior across the time points. The width of each path represents the proportion of individuals who change categories. The colors highlight the different categories of smoking: orange (T0/T1) and red (T2) are used to represent smoking and green non-smoking, respectively. At both follow-up points, more people had stopped smoking than started smoking. Non-smoking increased from T0 to T1 (7,582 to 7,614) and from T1 to T2 (7,614 to 7,644). Smoking decreased accordingly, from T0 to T1 (699 to 667) and from T1 to T2 (667 to 637)

Figure 2 visualizes the change in smoking status over time between the measurement points and shows that there was a slow decrease in the number of individuals who reported smoking over time. Of those who reported smoking in 2020, 17% had quit smoking by 2021, and an additional 18% had quit by 2022. However, some participants had started or resumed smoking at T1 and T2, resulting in a net relative reduction of 9% in smokers over the two years. Women more often changed smoking status between measuring points than men (see Additional Figures 1 and 2 ). More information about tobacco consumption is presented in Additional file 1 and Additional Table 5.

At T0, the group that reported severe psychological distress had a higher proportion of smokers than those with no psychological distress (13% absolute difference (CI 10%; 15%)) (Table 2). However, the differences in the number of smokers decreased slightly over time. At T0, participants between ≥40 and <60 years of age had the largest proportion of smokers compared to the youngest age group <30 years. Participants with children below 18 years at home had a lower proportion of smokers than those without (-2% (CI -3%; -1%)) at T0. Likewise, participants in home office/school (-4% (CI -5%; -4%)) and those with higher education (-5% (CI -6%; -4%)) had a lower prevalence of smokers compared to those not working from home and with lower education, respectively. In the multivariable analysis only one variable turned out significant over time, showing that those with high levels of psychological distress quitted smoking to a higher degree than those with lower levels of psychological distress (-2% (CI -3%; -1%).

**Table 2:** Adjusted linear mixed model for smoking (*n* = 21,846) presented on an absolute percentage scale (-100─100 %)

	Baseline	Time trend
	**coefficients (95% CI)**	**coefficients per year (95% CI)**
Male	0.51 (-0.25;1.27)	-0.08 (-0.42;0.25)
Years of age:		
18–29	0 (ref.)	0 (ref.)
30–39	5.71 (4.33;7.09)*	-0.60 (-1.31;0.12)
40–49	10.72 (9.32;12.12)*	-0.71 (-1.42;0.00)
50–59	11.35 (10.05;12.66)*	-0.05 (-0.70;0.60)
60–69	7.74 (6.31;9.16)*	-0.13 (-0.82;0.55)
≥ 70	0.80 (-0.86;2.47)	0.69 (-0.09;1.47)
Children <18 years at home	-2.04 (-3.03;-1.05)*	0.41 (-0.04;0.87)
Living alone	3.17 (2.17;4.18)*	-0.22 (-0.65;0.21)
Higher education	-5.03 (-5.82;-4.24)*	0.31 (-0.04;0.65)
Temporarily laid off	1.60 (0.12;3.08)	0.15 (-0.57;0.86)
Home office/study	-4.36 (-5.20;-3.53)*	0.35 (-0.02;0.73)
Economic worries	1.08 (-0.05;2.21)	-0.14 (-0.67;0.40)
Health worries	0.04 (-0.74;0.81)	0.31 (-0.04;0.65)
Psychological distress	12.82 (10.43;15.21)*	-1.74 (-2.85;-0.63)*
PA level:		
Low	0 (ref.)	0 (ref.)
Moderate	-3.22 (-4.14;-2.30)*	0.17 (-0.24;0.58)
High	-3.66 (-4.65;-2.67)*	0.30 (-0.15;0.74)
Hazardous drinking	3.96 (3.21;4.71)*	-0.32 (-0.65;0.01)
Constant/time	4.43 (2.79;6.06)	-0.55 (-1.34;0.25)

The table displays a linear mixed model regression analysis evaluating the associations between psychological distress and worries related to health and economy, pandemic measures, and personal situation (predictors) and adjusted for age and gender on tobacco smoking (dichotomized as non-smoking (0) or smoking (1)) at baseline and the predictors’ influence on changes in tobacco smoking (time trend) per year from baseline

*CI* Confidence interval

^*^ Significantly different from the reference group (*p* < 0.05)

Over the span of two years, both smokers and non-smokers experienced approximately the same number of instances and durations of being in quarantine or isolation (see Additional Table 6).

## Discussion

The findings of this large population cohort study provide valuable insights into smoking behaviors and associated factors during the COVID-19 pandemic. The proportion of smokers among the participants was 10% at T0 and 8% at T2. Higher smoking prevalence was observed among individuals with severe psychological distress, people over 40 years old, those not working from home, those with lower educational levels, lower PA levels, and individuals with hazardous alcohol use at T0/baseline. Throughout the COVID-19 pandemic, there was a slight reduction in the smoking rate among individuals with severe psychological distress.

The study showed that approximately 10% of the study sample were current smokers at baseline, which is lower than other reports from Norway that range from 12−19% [19] but higher than the estimate that 7% of the Norwegian population are daily smokers [20]. Public health surveys conducted in the same region as the present study showed a decline in the percentage of daily smokers from 9% in 2018 [21] to 7% in 2022 [22]. Our study did, however, not provide information about the volume of tobacco consumed at baseline, hence the study sample was not limited to daily smokers. At baseline, a higher percentage of smokers was reported among people with severe psychological distress, women, people over 40 years of age compared with those under 30 years, people not working from home, those with lower educational levels, those with lower levels of PA, and people with hazardous alcohol use.

Our study identified a notable net decrease in the number of smokers during the COVID-19 pandemic, similar to trends observed in other countries [9, 23, 24], despite some participants starting or resuming smoking during the observation period. Several factors likely contributed to this decline, including the association of smoking with severe COVID-19 outcomes, as smokers are at higher risk for viral and bacterial respiratory infections [25–27]. While some studies suggest a link between smoking and increased harm from COVID-19 [28, 29], a meta-analysis found limited evidence for that claim due to small sample sizes [30]. Nevertheless, the pandemic seems to have induced changes in tobacco consumption, driven by individual perceptions of the virus's harm. The crisis likely heightened health consciousness and motivated people to prioritize their health and well-being, leading to positive behavioral changes such as quitting smoking to reduce respiratory vulnerability [31–33]. Furthermore, the unique pandemic setting, characterized by lockdowns and social distancing measures, reduced smoking opportunities and exposure to smoking environments. The disruption of daily routines and increased focus on health-related behaviors during the pandemic created a window of opportunity for smokers to quit. This is supported by findings from a Finnish study, which indicated that a 500-meter increase in distance from home to a cigarette outlet was associated with a 16% increase in the odds of quitting smoking [34]. Another study reported that cigarette availability or the presence of other smokers predicted an odds ratio of 4.5 for lapsing during a quit attempt [35]. One might speculate that isolation and less availability of cigarettes could produce the opposite effect.

Our study revealed a higher prevalence of smokers among individuals experiencing severe psychological distress compared to those without distress, which is consistent with findings from national studies in Norway and other countries [36–39]. This observation is in line with previous research showing a strong link between mental health issues and smoking behavior [40, 41]. Several factors could explain this association. The pandemic itself may contribute to increased anxiety, depression, and reduced mental well-being, as observed in other crises [42]. Additionally, continuous media coverage and exposure to pandemic-related information, including rising case numbers and mortality rates, could have a further negative impact on respondents' mental health. Moreover, self-isolation and social distancing measures are likely to adversely affect mental well-being, leading to higher levels of anxiety and depression, as suggested in previous studies [43]. While individuals with the highest levels of psychological distress initially had a 13% higher prevalence of smoking than those with the lowest levels of psychological distress, the excess prevalence of smoking had reduced over the two years among people with the highest levels of psychological distress but was still 9% higher. The underlying reasons for this can probably not be deduced directly from our data. One hypothesis is that population level interventions targeting the management of psychological distress may have contributed to this improvement, helping individuals find other ways to regulate their emotions besides smoking. Another hypothesis suggests that population efforts to reduce smoking may have reached those with high levels of psychological distress particularly well.

Individuals in their forties to sixties had approximately a 10% higher smoking rate compared to both those under 30 years of age and those aged 70 and above. These cohort differences remained relatively stable across the two years of follow-up. For other factors such as education and PA, the time trends remained stable over time. The study also found that participants with children below 18 years at home smoked less than those without children. This finding aligns with previous research indicating that parenthood is associated with decreased smoking rates among adults [44]. School closures could also have reduced smoking behaviors among parents seeking to avoid smoking around children; in addition, for some, work- or commuting-related stress was potentially alleviated [24]. The desire to create a smoke-free environment for children and the role-modeling aspect of parental behavior may contribute to reduced smoking among individuals with children. The increased health consciousness during the pandemic, coupled with the desire to protect children from the harmful effects of secondhand smoke, may have further motivated parents to quit smoking or reduce their smoking habits.

People working or studying from home smoked less than those not working from home in our data. The home office/school setting may provide a more controlled environment that reduces exposure to social cues or stressors that typically trigger smoking. The absence of workplace smoking breaks or peer pressure to smoke may have contributed to lower smoking rates among individuals in this setting. The pandemic-induced shift to remote work and virtual learning may have created an environment that supported smoking cessation efforts among this group. Some studies have shown that smoking declines during short-term economic downturns/crises [45]. This was particularly found among heavy smokers. The authors’ hypothesized explanation for these findings is that declining work hours contributed to increased health investment [46].

Additionally, we found that participants with higher education accounted for fewer smokers than those with lower education. Research has shown that cigarette smoking is significantly associated with lower income [47, 48]. Education empowers individuals to acquire skills and general health knowledge, thereby increasing their awareness of healthy behaviors and preventive healthcare practices [49]. Thus, individuals with higher education may be more health-conscious and more aware of the health risks associated with smoking.

Our study found that living alone negatively affected smoking, suggesting that social isolation may contribute to increased smoking. This finding is consistent with previous research indicating that living alone is associated with higher smoking rates [50]. The absence of social support systems or accountability measures that exist in shared living arrangements could contribute to increased smoking among those living alone. The pandemic, with its associated restrictions and social distancing measures, may have intensified feelings of loneliness and isolation, potentially influencing smoking behaviors.

Furthermore, the results of our study, surveying a representative sample of adults in Bergen, Norway, displayed a higher percentage of smokers among people with hazardous alcohol use. Both alcohol and tobacco are addictive substances that can have significant effects on an individual’s health, behavior, and well-being. It is not uncommon for individuals who are addicted to one substance to be more likely to use or become addicted to another [51, 52]. Research suggests that tobacco can enhance the rewarding effects of alcohol, and vice versa [6], and that their consumption can as such increase cravings for each other [53]. Tobacco smoking in conjunction with hazardous alcohol consumption is of particular concern because it can increase the risk of several cancer types, as well as cardiovascular diseases and respiratory problems [54–56]. The links between stress, depression, anxiety, and tobacco use are also contributing factors for comorbidity [40]. Recent studies showed that individuals with psychological distress had higher levels of alcohol consumption during COVID-19 [57, 58].

The analysis of smoking trends in our study showed that individuals with severe psychological distress exhibited a notable decline in smoking during the two years of the COVID-19 pandemic. This aligns with findings from other studies, where smokers with severe psychological distress were significantly more likely to report intentions to quit smoking and seek counseling than smokers without severe psychological distress [9, 59, 60]. This decline is particularly noteworthy considering a major economic crisis that led to widespread job loss, resulting in four times higher unemployment and lower psychological and physical well-being during the COVID-19 lockdown in Norway [61, 62]. The enormous government pandemic-relief spending [63, 64] and worries about the dangerous respiratory virus might have deterred the increase in cigarette use, as reported in many high-income countries [24, 28]. The government introduced several measures to support individuals and businesses during the pandemic, such as compensation schemes, training programs, and loans [65]. These measures could have alleviated financial stress, which might indirectly affect smoking behavior. A similar reduction in the prevalence of smoking was also observed following other major crises in England (the Great Recession from 2001–2013) and Iceland (the economic collapse in 2007−2009) that resulted in an increased rate of smoking cessation, even among people with mental health problems [62, 66]. Additionally, studies suggest that when people have less money to spend, they may be less likely to purchase cigarettes, which could lead to a decrease in smoking [67, 68].

Our study identified a decrease in smoking prevalence over time, particularly among specific groups such as those with severe psychological distress. This suggests that crises, such as the pandemic, can serve as catalysts for positive behavioral changes, including smoking cessation. Furthermore, the study emphasized the link between mental health and smoking, indicating a higher prevalence of smoking among individuals experiencing severe psychological distress. The findings underscore the need for targeted interventions that address both smoking cessation and mental health support, especially during crises. Moreover, the study identified demographic factors such as educational level, parenthood, living arrangements, and alcohol use as significant contributors to smoking behaviors. Tailoring interventions based on these factors can be crucial in developing effective strategies for smoking cessation.

### Strengths and limitations

The present study has several strengths, notably the ability to conduct highly precise and statistically powerful analyses due to the large sample size. Furthermore, the yearly follow-ups of participants offered valuable insights into changes occurring over the pandemic era. Although a considerable proportion of participants dropped out during these follow-ups, we checked background factors in each of the follow-ups, which were generally quite comparable. A 36% response rate at T0 is deemed adequate for an online survey [69]. A meta-analysis examining 39 comparative results determined that the unweighted average response rate for online surveys was 34% [70]. The follow-up rates at T1 and T2, which were 63% and 37% respectively, align with response rates reported in other longitudinal online COVID-19 health surveys [71, 72]. The large sample size might lead to statistically significant findings without necessarily having practical importance and relevance. While the sample was randomly selected from a broad population, variations in response within different strata of the population could result in our cohort not being entirely representative of the source population, potentially limiting generalizability. Although recruitment to the study was based on random sampling, the electronic approach could have influenced the results and potentially excluded individuals with lower digital literacy. This exclusion may affect the generalizability of conclusions in terms of health-related behaviors and pandemic awareness. Excluding individuals with lower digital literacy will result in limited insights into the impact of socioeconomic status on smoking behaviors during the pandemic and overemphasize behaviors and characteristics of individuals with higher digital literacy. People who smoke tobacco might have higher non-response rates than their non-smoking peers, but the Sankey analyses we report only include those with complete data at both baseline and the two follow-ups. This approach reduces the risk of attrition bias but may limit generalizability.

Information about smoking habits relied on self-reported information. Self-reported tobacco consumption often faces the issue of underreporting [73]. Social desirability bias can lead individuals to respond in a way that presents them more favorably in social contexts. Consequently, people may downplay their tobacco consumption to provide answers they perceive as socially acceptable, even if these responses are inaccurate. Our study discusses associations between smoking and various factors, including mental health, remote work, and education. Underreporting may introduce bias into these associations, potentially affecting the validity of conclusions drawn about the relationships between smoking and other variables. However, as our data were collected through email and short text messages, they may be more credible in this context compared to data collected through interviews or phone calls. In line with this reasoning, respondents may feel less exposed when answering behavioral questions in written form, potentially reducing the influence of social desirability bias. It should be noted that no information was available regarding the extent non-smokers at baseline were former smokers.

## Conclusions

The findings of this large population cohort study conducted during the COVID-19 pandemic in Bergen, Norway, revealed a higher prevalence of smoking among individuals with high levels of psychological distress, women, older age groups (40−60 years), and those who were not working from home, had lower education, had sedentary lifestyles, or engaged in hazardous alcohol use at baseline. However, the association between psychological distress and smoking was reduced, showing a gradual decrease in the prevalence of smoking among individuals with severe psychological distress as time progressed during the COVID-19 pandemic.

### Supplementary Information

**Additional file 1.** Supplementary information about tobacco consumption. **Additional Table 1.** Age, gender, and educational level of non-respondents at follow-ups assessed with logistic regression utilizing odds ratios and 95% confidence intervals. **Additional Table 2. **Background characteristics of participants per age group, April 2020. **Additional Table 3. **Background characteristics of participants per age group, January 2021. **Additional Table 4. **Background characteristics of participants per age group, January 2022. **Additional Table 5.** Change of nicotine consumption in relation to age for all in addition to women and men, separately.**Additional Table 6. **Illness periods* over the two years**. **Additional Figure 1.** Sankey diagram of change in smoking behavior per gender, women. **Additional Figure 2.** Sankey diagram of change in smoking behavior per gender, men.

## Data Availability

The datasets generated and/or analyzed during the current study are not publicly available due to privacy restrictions but are available from the corresponding author on reasonable request.

## Abbreviations

AUDIT-C: Alcohol Use Disorder Identification Test - Consumption
CI: Confidence interval
COVID-19: Coronavirus disease-2019
EUR: Euro
IPAQ-SF: International Physical Activity Questionnaire - Short Form
MET: Metabolic equivalent of task
NOK: Norwegian krone
PA: Physical activity
SCL-10: Symptom Checklist ten-items

## Footnotes

1 In Norway, the common contact register is a system that contains the contact information of citizens and residents. The register is used by Norwegian public authorities to send messages and documents to individuals. The Norwegian Digitalization Agency is responsible for the contact information in the common contact register, which contains mobile phone numbers, postal addresses, and e-mail addresses.
