# Supplementary material for: The association of psychological distress and economic and health worries with tobacco smoking behavior during the COVID-19 pandemic: a two-year longitudinal cohort study
Source: BMC Public Health. 2024 Feb 5;24:375. doi: 10.1186/s12889-024-17943-x (PMC10840189; doi:10.1186/s12889-024-17943-x)
Supplement: Supplementary file 1 — Additional file 1. Supplementary information about tobacco consumption. Additional Table 1. Age, gender, and educational level of non-respondents at follow-ups assessed with logistic regression utilizing odds ratios and 95% confidence intervals. Additional Table 2. Background characteristics of participants per age group, April 2020. Additional Table 3. Background characteristics of participants per age group, January 2021. Additional Table 4. Background characteristics of participants per age group, January 2022. Additional Table 5. Change of nicotine consumption in relation to age for all in addition to women and men, separately.Additional Table 6. Illness periods* over the two years**. Additional Figure 1. Sankey diagram of change in smoking behavior per gender, women. Additional Figure 2. Sankey diagram of change in smoking behavior per gender, men. [file 12889_2024_17943_MOESM1_ESM.docx]

# Supplementary material

**Additional Table 1.** Age, gender, and educational level of non-respondents at follow-ups assessed with logistic regression utilizing odds ratios and 95% confidence intervals

| Variables | Odds ratio (95% CI) |
| --- | --- |
| Female | 1.0 (ref.) |
| Male | 1.19 (1.13;1.25) |
| Years of age: |  |
| 18–29 | 1.0 (ref.) |
| 30–39 | 0.71 (0.64;0.78) |
| 40–49 | 0.59 (0.53;0.65) |
| 50–59 | 0.42 (0.38;0.46) |
| 60–69 | 0.32 (0.29;0.35) |
| ≥ 70 | 0.35 (0.31;0.38) |
| Education: |  |
| Primary school | 1.0 (ref.) |
| High school | 0.83 (0.74;0.92) |
| University ≤ 3 years | 0.68 (0.61;0.76) |
| University > 3 years | 0.58 (0.52;0.64) |

*CI* Confidence interval

**Additional Table 2.** Background characteristics of participants per age group, April 2020

| **Age** | **18–29** | **30–39** | **40–49** | **50–59** | **60–69** | **70+** | **Total**  ***n* (%)** |
| --- | --- | --- | --- | --- | --- | --- | --- |
| ***n*** | 3234 (13%) | 3957 (16%) | 4520 (18%) | 5115 (21%) | 4450 (18%) | 3638 (15%) | 24,914 (100%) |
| Gender (women) | 2083 (64%) | 2370 (60%) | 2656 (59%) | 2939 (57%) | 2296 (52%) | 1735 (48%) | 14,079 (57%) |
| Primary school | 404 (13%) | 149 (4%) | 173 (4%) | 268 (5%) | 390 (9%) | 472 (13%) | 1856 (8%) |
| High school | 1153 (36%) | 724 (18%) | 892 (20%) | 1586 (31%) | 1432 (32%) | 1191 (33%) | 6978 (28%) |
| University ≤ 3 years | 838 (26%) | 1012 (26%) | 1073 (24%) | 1228 (24%) | 986 (22%) | 811 (23%) | 5948 (24%) |
| University > 3 years | 822 (26%) | 2044 (52%) | 2356 (52%) | 2005 (39%) | 1618 (37%) | 1123 (31%) | 9968 (40%) |
| Adjusted income (EUR) * |  |  |  |  |  |  |  |
| 0–25,000 | 1031 (37%) | 521 (14%) | 465 (11%) | 363 (8%) | 245 (6%) | 380 (13%) | 3005(14%) |
| 25,000–50,000 | 1076 (38%) | 1867 (49%) | 2192 (51%) | 1769 (38%) | 1359 (36%) | 1432 (50%) | 9695 (44%) |
| >50,000 | 708 (25%) | 1404 (37%) | 1612 (38%) | 2515 (54%) | 2201 (58%) | 1045 (37%) | 9485 (43%) |
| Persons in household |  |  |  |  |  |  |  |
| 1 | 460 (14%) | 581 (15%) | 493 (11%) | 849 (17%) | 1214 (28%) | 1452 (43%) | 5049 (21%) |
| 2 | 1127 (35%) | 847 (22%) | 589 (13%) | 1573 (32%) | 2178 (51%) | 1550 (46%) | 7864 (33%) |
| 3–4 | 1185 (37%) | 1856 (47%) | 2204 (50%) | 2071 (42%) | 791 (19%) | 329 (10%) | 8436 (35%) |
| 5+ | 421 (13%) | 624 (16%) | 1150 (26%) | 484 (10%) | 80 (2%) | 50 (1%) | 2809 (12%) |
| Employment | 2122 (66%) | 3402 (86%) | 4036 (89%) | 4494 (88%) | 2486 (56%) | 229 (6%) | 16,769 (67%) |
| Student/school | 1566 (48%) | 246 (6%) | 104 (2%) | 32 (1%) | 7 (0%) | 3 (0%) | 1958 (8%) |
| Placed in quarantine | 729 (23%) | 639 (16%) | 678 (15%) | 723 (14%) | 668 (15%) | 598 (16%) | 4035 (16%) |
| Temporarily laid-off | 495 (15%) | 375 (9%) | 344 (8%) | 394 (8%) | 217 (5%) | 20 (1%) | 1845 (8%) |
| Home office/study | 2154 (67%) | 2579 (65%) | 3055 (68%) | 2825 (55%) | 1463 (33%) | 153 (4%) | 12,229 (49%) |
| COVID-19 symptoms | 271 (8%) | 335 (8%) | 365 (8%) | 318 (6%) | 162 (4%) | 80 (2%) | 1531 (6%) |
| Worries | 2135 (66%) | 2367 (60%) | 2398 (53%) | 2763 (54%) | 1834 (41%) | 1137 (31%) | 12,634 (51%) |
| Worries related to economy | 940 (29%) | 954 (24%) | 843 (19%) | 839 (16%) | 357 (8%) | 81 (2%) | 4014 (16%) |
| Health-related worries | 1782 (55%) | 1944 (49%) | 2070 (46%) | 2429 (47%) | 1663 (37%) | 1091 (30%) | 10,979 (44%) |
| Psychological distress | 1311 (41%) | 1119 (28%) | 879 (19%) | 787 (15%) | 487 (11%) | 289 (8%) | 4872 (20%) |
| Smoking | 161 (5%) | 295 (7%) | 512 (11%) | 723 (14%) | 509 (11%) | 202 (6%) | 2402 (10%) |
| PA level |  |  |  |  |  |  |  |
| Low | 895 (28%) | 1107 (29%) | 1108 (26%) | 1034 (22%) | 799 (21%) | 702 (27%) | 5645 (25%) |
| Moderate | 1343 (43%) | 1644 (43%) | 1860 (44%) | 2055 (44%) | 1664 (44%) | 1148 (44%) | 9714 (44%) |
| High | 907 (29%) | 1074 (28%) | 1271 (30%) | 1547 (33%) | 1327 (35%) | 769 (29%) | 6895 (31%) |
| Harmful drinking | 2172 (67%) | 2117 (54%) | 2474 (55%) | 2723 (54%) | 2335 (53%) | 1296 (37%) | 13,117 (53%) |

* The adjusted income is the household income divided by the personal index. The personal index is calculated as 1 for the first adult, 0.7 per other adult household member, and 0.5 per child. The adjusted income was converted to Euros.

**Additional Table 3.** Background characteristics of participants per age group, January 2021

| **Age** | **18–29** | **30–39** | **40–49** | **50–59** | **60–69** | **70+** | **Total**  ***n* (%)** |
| --- | --- | --- | --- | --- | --- | --- | --- |
| ***n*** | 1641 (9%) | 2517 (15%) | 3139 (18%) | 3779 (22%) | 3499 (20%) | 2737 (16%) | 17,312 (100%) |
| Gender (women) | 1069 (65%) | 1539 (61%) | 1872 (60%) | 2194 (58%) | 1784 (51%) | 1303 (48%) | 9761 (56%) |
| Primary school | 169 (12%) | 70 (3%) | 102 (3%) | 167 (5%) | 270 (8%) | 298 (11%) | 1076 (7%) |
| High school | 462 (32%) | 372 (16%) | 542 (19%) | 1108 (31%) | 1072 (32%) | 847 (32%) | 4403 (27%) |
| University ≤ 3 years | 393 (27%) | 590 (26%) | 689 (24%) | 886 (25%) | 779 (23%) | 617 (23%) | 3954 (24%) |
| University > 3 years | 411 (29%) | 1282 (55%) | 1594 (54%) | 1453 (40%) | 1259 (37%) | 884 (33%) | 6883 (42%) |
| Adjusted income (EUR) * |  |  |  |  |  |  |  |
| 0–25,000 | 421 (33%) | 230 (10%) | 260 (9%) | 249 (7%) | 176 (6%) | 256 (12%) | 1592 (11%) |
| 25,000–50,000 | 475 (37%) | 1078 (48%) | 1431 (51%) | 1244 (37%) | 1045 (35%) | 1086 (51%) | 6359 (43%) |
| >50,000 | 372 (29%) | 937 (42%) | 1114 (40%) | 1838 (55%) | 1728 (59%) | 808 (38%) | 6797 (46%) |
| Persons in household |  |  |  |  |  |  |  |
| 1 | 225 (16%) | 378 (16%) | 340 (12%) | 635 (18%) | 895 (27%) | 1041 (42%) | 3514 (22%) |
| 2 | 523 (37%) | 498 (22%) | 386 (13%) | 1132 (32%) | 1712 (52%) | 1191 (48%) | 5442 (34%) |
| 3–4 | 500 (35%) | 1067 (46%) | 1430 (49%) | 1426 (40%) | 607 (19%) | 241 (10%) | 5271 (33%) |
| 5+ | 174 (12%) | 358 (16%) | 738 (26%) | 348 (10%) | 61 (2%) | 31 (1%) | 1710 (11%) |
| Employment | 991 (60%) | 2049 (81%) | 2666 (85%) | 3198 (85%) | 1885 (54%) | 166 (6%) | 10,955 (63%) |
| Student/school | 645 (39%) | 105 (4%) | 65 (2%) | 20 (1%) | 4 (0%) | 2 (0%) | 841 (5%) |
| Placed in quarantine | 356 (22%) | 416 (17%) | 477 (15%) | 541 (14%) | 534 (15%) | 484 (18%) | 2808 (16%) |
| Temporarily laid-off | 210 (13%) | 187 (7%) | 211 (7%) | 289 (8) | 167 (5%) | 14 (1%) | 1078 (6%) |
| Home office/study | 1084 (66%) | 1673 (66%) | 2136 (68%) | 2124 (56%) | 1148 (33%) | 121 (4%) | 8286 (48%) |
| COVID-19 symptoms | 131 (8%) | 210 (8%) | 245 (8%) | 232 (6%) | 118 (3%) | 51 (2%) | 987 (6%) |
| Worries | 931 (57%) | 1384 (55%) | 1526 (49%) | 1953 (52%) | 1377 (39%) | 808 (30%) | 7979 (46%) |
| Worries related to economy | 388 (24%) | 521 (21%) | 492 (16%) | 575 (15%) | 265 (8%) | 63 (2%) | 2304 (13%) |
| Health-related worries | 788 (48%) | 1166 (46%) | 1318 (42%) | 1706 (45%) | 1247 (36%) | 770 (28%) | 6995 (40%) |
| Psychological distress | 577 (35%) | 675 (27%) | 553 (18%) | 546 (14%) | 358 (10%) | 187 (7%) | 2896 (17%) |
| Smoking | 53 (3%) | 138 (5%) | 305 (10%) | 479 (13%) | 371 (11%) | 143 (5%) | 1489 (9%) |
| PA level |  |  |  |  |  |  |  |
| Low | 421 (29%) | 668 (29%) | 730 (26%) | 729 (22%) | 588 (20%) | 478 (24%) | 3614 (24%) |
| Moderate | 630 (43%) | 1031 (44%) | 1277 (45%) | 1518 (45%) | 1340 (45%) | 913 (46%) | 6709 (45%) |
| High | 398 (27%) | 642 (27%) | 855 (30%) | 1117 (33%) | 1042 (35%) | 599 (30%) | 4653 (31%) |
| Harmful drinking | 980 (68%) | 1303 (56%) | 1674 (57%) | 1969 (55%) | 1814 (54%) | 1002 (38%) | 8742 (54%) |

* The adjusted income is the household income divided by the personal index. The personal index is calculated as 1 for the first adult, 0.7 per other adult household member, and 0.5 per child. The adjusted income was converted to Euros.

**Additional Table 4.** Background characteristics of participants per age group, January 2022

| **Age** | **18–29** | **30–39** | **40–49** | **50–59** | **60–69** | **70+** | **Total**  ***n* (%)** |
| --- | --- | --- | --- | --- | --- | --- | --- |
| ***n*** | 806 (8%) | 1361 (13%) | 1754 (17%) | 2347 (23%) | 2269 (22%) | 1731 (17%) | 10,268 (100%) |
| Gender (women) | 554 (69%) | 867 (64%) | 1090 (62%) | 1383 (59%) | 1165 (51%) | 837 (48%) | 5896 (57%) |
| Primary school | 84 (11%) | 36 (3%) | 50 (3%) | 102 (5%) | 163 (7%) | 164 (10%) | 599 (6%) |
| High school | 226 (31%) | 204 (16%) | 293 (18%) | 639 (28%) | 653 (30%) | 527 (31%) | 2542 (26%) |
| University ≤ 3 years | 205 (28%) | 305 (24%) | 389 (23%) | 535 (24%) | 492 (22%) | 407 (24%) | 2333 (24%) |
| University > 3 years | 222 (30%) | 717 (57%) | 926 (56%) | 982 (43%) | 894 (41%) | 584 (35%) | 4325 (44%) |
| Adjusted income (EUR) * |  |  |  |  |  |  |  |
| 0–25,000 | 209 (32%) | 131 (11%) | 139 (9%) | 143 (7%) | 101 (5%) | 153 (11%) | 876 (10%) |
| 25,000–50,000 | 261 (40%) | 583 (48%) | 823 (52%) | 761 (36%) | 682 (35%) | 694 (50%) | 3804 (43%) |
| >50,000 | 175 (27%) | 508 (42%) | 627 (39%) | 1199 (57%) | 1152 (60%) | 544 (39%) | 4205 (47%) |
| Persons in household |  |  |  |  |  |  |  |
| 1 | 129 (18%) | 216 (17%) | 201 (12%) | 418 (19%) | 569 (27%) | 655 (41%) | 2188 (23%) |
| 2 | 284 (39%) | 273 (22%) | 227 (14%) | 710 (32%) | 1133 (53%) | 757 (47%) | 3384 (35%) |
| 3–4 | 228 (31%) | 591 (47%) | 795 (48%) | 888 (40%) | 405 (19%) | 172 (11%) | 3079 (32%) |
| 5+ | 85 (12%) | 173 (14%) | 418 (25%) | 199 (9%) | 36 (2%) | 24 (1%) | 935 (10%) |
| Employment | 508 (63%) | 1121 (82%) | 1505 (86%) | 2012 (86%) | 1231 (54%) | 101 (6%) | 6478 (63%) |
| Student/school | 331 (41%) | 68 (5%) | 39 (2%) | 14 (1%) | 2 (0%) | 2 (0%) | 456 (4%) |
| Placed in quarantine | 167 (21%) | 209 (15%) | 270 (15%) | 348 (15%) | 354 (16%) | 281 (16%) | 1629 (16%) |
| Temporarily laid-off | 105 (13%) | 96 (7%) | 96 (5%) | 170 (7%) | 109 (5%) | 5 (0%) | 581 (6%) |
| Home office/study | 551 (68%) | 901 (66%) | 1189 (68%) | 1356 (58%) | 771 (34%) | 73 (4%) | 4841 (47%) |
| COVID-19 symptoms | 64 (8%) | 114 (8%) | 143 (8%) | 147 (6%) | 90 (4%) | 37 (2%) | 595 (6%) |
| Worries | 469 (58%) | 720 (53%) | 851 (49%) | 1204 (51%) | 887 (39%) | 482 (28%) | 4613 (45%) |
| Worries related to economy | 197 (24%) | 279 (21%) | 268 (15%) | 326 (14%) | 172 (8%) | 33 (2%) | 1275 (12%) |
| Health-related worries | 387 (48%) | 585 (43%) | 745 (43%) | 1074 (46%) | 800 (35%) | 461 (27%) | 4052 (39%) |
| Psychological distress | 288 (36%) | 363 (27%) | 314 (18) | 344 (15%) | 249 (11%) | 114 (7%) | 1672 (16%) |
| Smoking | 26 (3%) | 68 (5%) | 156 (9%) | 265 (11%) | 198 (9%) | 83 (5%) | 796 (8%) |
| PA level |  |  |  |  |  |  |  |
| Low | 226 (31%) | 358 (28%) | 440 (27%) | 455 (21%) | 387 (20%) | 310 (24%) | 2176 (24%) |
| Moderate | 329 (44%) | 573 (45%) | 711 (44%) | 972 (46%) | 886 (45%) | 607 (46%) | 4078 (45%) |
| High | 185 (25%) | 341 (27%) | 474 (29%) | 694 (33%) | 690 (35%) | 393 (30%) | 2777 (31%) |
| Harmful drinking | 509 (69%) | 700 (55%) | 941 (57%) | 1221 (54%) | 1177 (54%) | 664 (40%) | 5212 (53%) |

* The adjusted income is the household income divided by the personal index. The personal index is calculated as 1 for the first adult, 0.7 per other adult household member, and 0.5 per child. The adjusted income was converted to Euros.

**Additional Table 5.** Change of nicotine consumption in relation to age for all in addition to women and men, separately

| Age | 18-29 | 30-39 | 40-49 | 50-59 | 60-69 | 70+ | Total |
| --- | --- | --- | --- | --- | --- | --- | --- |
| BASELINE |  |  |  |  |  |  |  |
| Increased nicotine consumption (both) | 68 (11%) | 93 (16%) | 164 (28%) | 161 (27%) | 87 (15%) | 19 (3%) | 592 (13%) |
| Women | 33 (9%) | 62 (16%) | 109 (29) | 110 (29%) | 51 (13%) | 13 (3%) | 378 (18%) |
| Men | 35 (16%) | 31 (14%) | 55 (26%) | 51 (24%) | 36 (17%) | 6 (3%) | 214 (9%) |
| Decreased nicotine consumption (both) | 41 (8%) | 67 (13%) | 105 (20%) | 141 (27%) | 112 (21%) | 60 (11%) | 526 (12%) |
| Women | 15 (5%) | 34 (12%) | 61 (22%) | 75 (27%) | 57 (21%) | 36 (13%) | 278 (13%) |
| Men | 26 (10%) | 33 (13%) | 44 (18%) | 66 (27%) | 55 (22%) | 24 (10%) | 248 (11%) |
| 1-YEAR FOLLOW-UP |  |  |  |  |  |  |  |
| Increased nicotine consumption (both) | 24 (8%) | 47 (15%) | 76 (25%) | 88 (29%) | 54 (18%) | 17 (6%) | 306 (21%) |
| Women | 13 (7%) | 29 (15%) | 52 (26%) | 53 (27%) | 37 (19%) | 13 (7%) | 197 (23%) |
| Men | 11 (10%) | 18 (17%) | 24 (22%) | 35 (32%) | 17 (16%) | 4 (4%) | 109 (18%) |
| Decreased nicotine consumption (both) | 7 (2%) | 27 (10%) | 44 (16%) | 85 (30%) | 81 (29%) | 39 (14%) | 283 (19%) |
| Women | 6 (4%) | 16 (10%) | 27 (17%) | 51 (32%) | 39 (25%) | 20 (13%) | 159 (19%) |
| Men | 1 (1%) | 11 (9%) | 17 (14%) | 34 (27%) | 42 (34%) | 19 (15%) | 124 (20%) |
| 2-YEAR FOLLOW-UP |  |  |  |  |  |  |  |
| Increased nicotine consumption (both) | 8 (7%) | 18 (15%) | 31 (26%) | 35 (30%) | 18 (15%) | 8 (7%) | 118 (15%) |
| Women | 4 (5%) | 12 (16%) | 24 (32%) | 19 (25%) | 10 (13%) | 6 (8%) | 75 (16%) |
| Men | 4 (9%) | 6 (14%) | 7 (16%) | 16 (37%) | 8 (19%) | 2 (5%) | 43 (14%) |
| Decreased nicotine consumption (both) | 4 (3%) | 11 (7%) | 34 (22%) | 49 (32%) | 38 (25%) | 16 (11%) | 152 (20%) |
| Women | 3 (3%) | 3 (3%) | 24 (24%) | 35 (36%) | 22 (22%) | 11 (11%) | 98 (21%) |
| Men | 1 (2%) | 8 (15%) | 10 (19%) | 14 (26%) | 16 (30%) | 5 (9%) | 54 (18%) |

The table presents population weighted estimates (age, gender, education) for percentages.

**Additional Table 6.** Illness periods* over the two years**

|  | Smokers  *n* (%) | Non-smokers  *n* (%) | Total population  *n* (%) |
| --- | --- | --- | --- |
| Times in quarantine/isolation |  |  |  |
| 0 | 310 (41%) | 3252 (36%) | 3562 (36%) |
| 1−2 | 321 (43%) | 4261 (47%) | 4582 (47%) |
| ≥3 | 120 (16%) | 1563 (17%) | 1683 (17%) |
| Days in quarantine/isolation |  |  |  |
| <5 | 443 (59%) | 5019 (55%) | 5462 (55%) |
| 5−15 | 225 (30%) | 3078 (34%) | 3303 (34%) |
| 16−30 | 65 (9%) | 781 (9%) | 846 (9%) |
| >30 | 23 (3%) | 217 (2%) | 240 (2%) |
| Times with respiratory infection |  |  |  |
| 0 | 304 (40%) | 2741 (30%) | 3045 (31%) |
| 1−2 | 309 (41%) | 4085 (45%) | 4394 (45%) |
| ≥3 | 146 (19%) | 2218 (25%) | 2364 (24%) |
| Times with COVID-19 infection*** |  |  |  |
| 0 | 431 (55%) | 4151 (45%) | 4582 (46%) |
| 1 | 321 (41%) | 4777 (51%) | 5098 (51%) |
| ≥2 | 29 (4%) | 359 (4%) | 388 (4%) |

* Estimates of cumulative number

** Based on T2, smaller n

*** Certain or probable


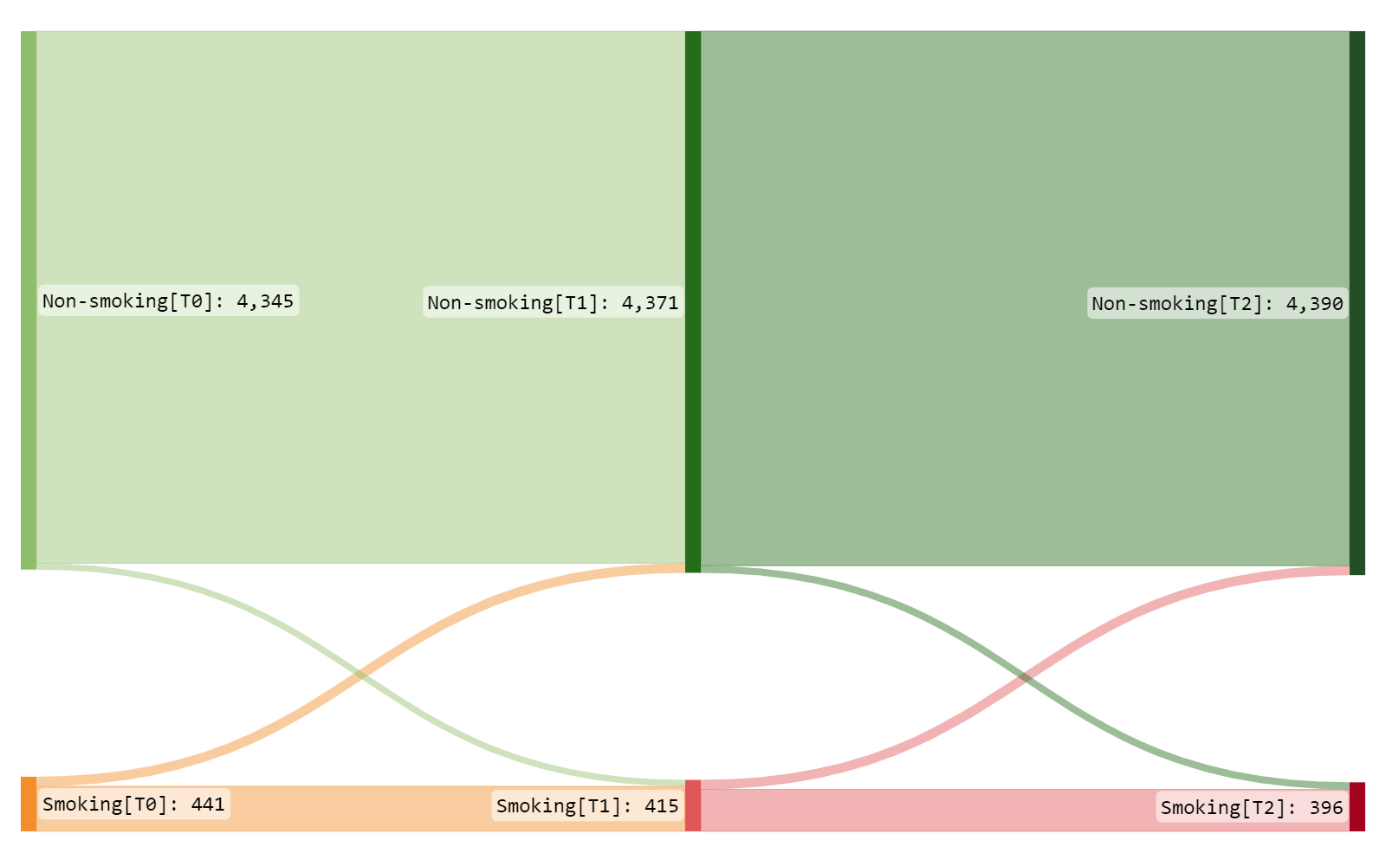


**Additional Figure 1.** Sankey diagram of change in smoking behavior per gender, women


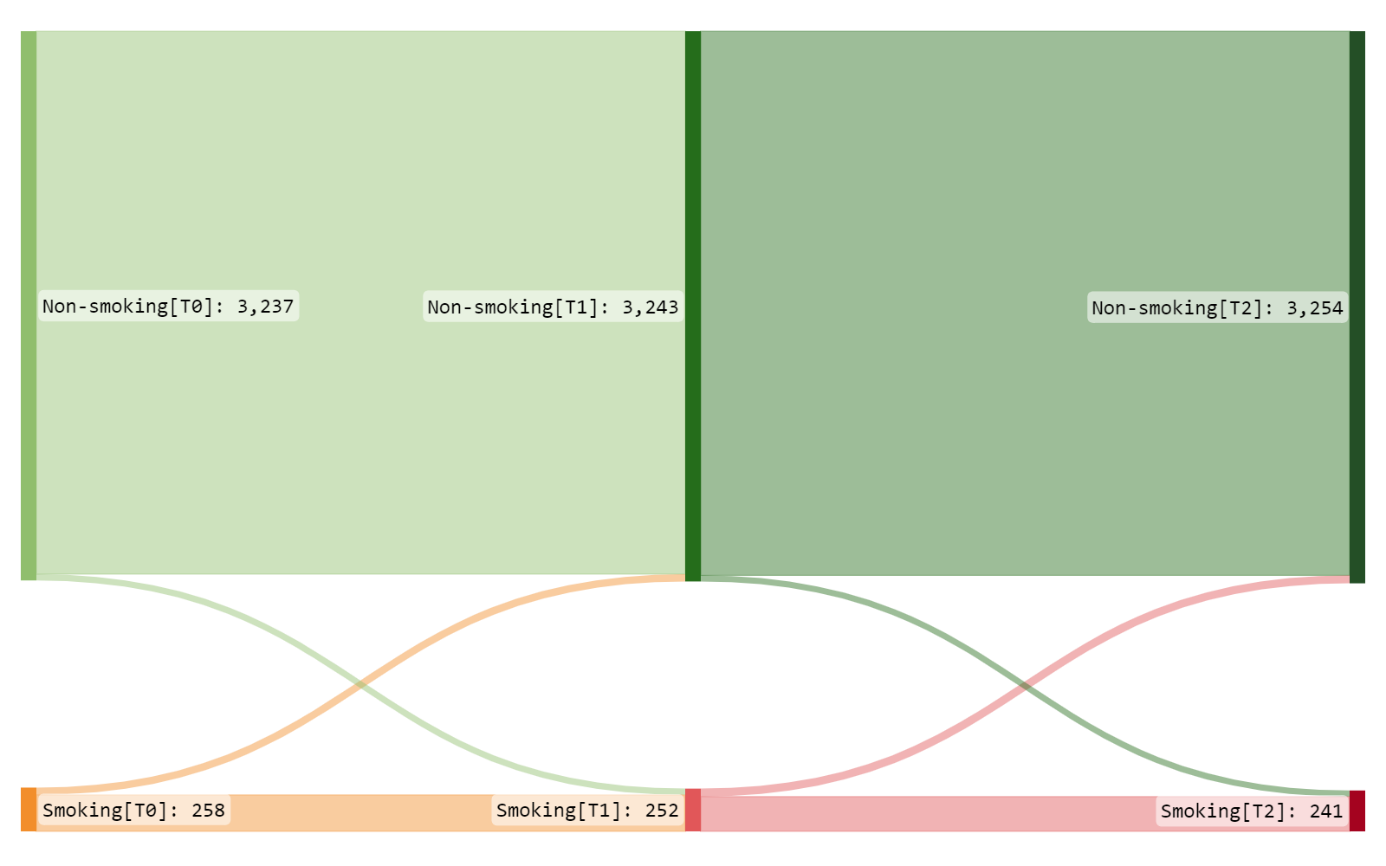


**Additional Figure 2.** Sankey diagram of change in smoking behavior per gender, men

**Additional File 1.** Supplementary information about tobacco consumption

Among those who were smoking, self-reported changes differed between groups. In the younger age groups (<50 years of age), more of those who used tobacco increased their tobacco consumption compared to those who reduced it. Conversely, particularly in the age group of 60 years and above, more individuals reported a decrease in their tobacco consumption than an increase (see Additional Table 5). Among those who reported smoking, more women than men increased their tobacco use. The mean number of cigarettes smoked per day among smokers did not change from 2021 (10.13 (SD 0.18; 95% 9.78;10.48)) to 2022 (9.97 (SD 0.24; 95% 9.49;10.44)) (t(2053) = 0.54, p > 0.5). Among the people who reported smoking at T1, 36% were often or very often worried about the harmful effects of smoking, 46% were sometimes worried, and 18% were rarely or never worried. Furthermore, 59% considered stopping completely, 27% wanted to reduce their smoking, and 14% did not want to reduce their smoking. Regarding the desire for help, 29% wanted assistance, 40% did not want help, and 32% were uncertain. Only 15% reported smoking indoors.

# Appendix

The smoking variables of the questionnaires consisted of the following questions:

T0 (2020)

**Do you currently smoke cigarettes or use snus?**

(1) ❑ Yes

(2) ❑ No

**Have you used more or less snus in the last month than the period before?**

(1) ❑ Have not used snus in the last month

(2) ❑ Have used less snus in the last month than before

(3) ❑ Snus usage has not changed much in the last month

(4) ❑ Have used more snus in the last month than before

**Have you smoked more or less in the last month than the period before?**

(1) ❑ Have not smoked in the last month

(2) ❑ Have smoked less in the last month than before

(3) ❑ Have smoked relatively unchanged in the last month

(4) ❑ Have smoked more in the last month than before

**Do you smoke indoors?**

(1) ❑ Yes

(2) ❑ No

(3) ❑ Not applicable

T1 (2021)

**Do you currently smoke cigarettes?**

(1) ❑ Yes

(2) ❑ No

(If Yes:)

**Have you smoked more or less in the last month than the period before?**

(1) ❑ Have smoked less in the last month than before

(2) ❑ Have smoked relatively unchanged in the last month

(3) ❑ Have smoked more in the last month than before

**How many cigarettes do you usually smoke per day?**

___ per day

**Check only one description that best fits you:**

❑ I get breathless only when I exercise hard (0)

❑ I get short of breath when I hurry on flat ground or uphill (1)

❑ I am slower than most people my age on flat ground, or I have to stop due to breathlessness when walking at my own pace on flat ground (2)

❑ I have to stop to catch my breath after 100 meters of walking, or after a few minutes at my own pace on flat ground (3)

❑ I am so breathless that I can't leave the house, or I get breathless when dressing and undressing (4)

**Are you concerned about the harmful effects of smoking?**

❑ Never

❑ Rarely

❑ Occasionally

❑ Often

❑ Very often

**Would you consider changing your smoking habits?**

❑ No

❑ Yes, reduce

❑ Yes, quit completely

❑ Yes, increase

**Do you smoke indoors?**

(1) ❑ Yes

(2) ❑ No

(3) ❑ Not applicable

**Would you like help to reduce or quit smoking?**

(1) ❑ Yes

(2) ❑ No

(3) ❑ Uncertain

T2 (2022)

**Do you currently smoke cigarettes?**

(1) ❑ Yes

(2) ❑ No

(If Yes:)

**Have you smoked more or less in the last month than the period before?**

(1) ❑ Have smoked less in the last month than before

(2) ❑ Have smoked relatively unchanged in the last month

(3) ❑ Have smoked more in the last month than before

**How many cigarettes do you usually smoke per day?**

___ per day

The AUDIT-C consists of the following three questions:

1. How often did you have a drink containing alcohol in the past year?

Answer: Never (score 0), Monthly or less (score 1), 2–4 times per month (score 2), 2–3 times per week (score 3), 4+ times per week (score 4)

One unit of alcohol is equivalent to one small bottle of beer, one small can of beer, a glass of wine, or one drink.

1. How many units of alcohol do you drink on a typical day when you are drinking?

Answer: 1–2 (score 0), 3–4 (score 1), 5–6 (score 2), 7–9 (score 3), 10+ (score 4)

1. How often do you drink 6 or more units of alcohol on the same occasion?

Answer: Never (score 0), Less than monthly (score 1), Monthly (score 2), Weekly (score 3), Daily or almost daily (score 4)

The variable “health-related worries” consisted of the following questions (with the response alternatives: “Strongly agree”, “Agree”, and “Disagree”):

1. I have become scared and anxious (worried) that the infection will affect some of my loved ones.
2. I have become scared and anxious (worried) that the infection will affect me.
3. I have become scared and anxious (worried) that the infection will affect some of the elderly members of the family.

The variable “economic worries” consists of the following questions with the response alternatives: “Strongly agree”, “Agree”, and “Disagree”):

1. I fear (am worried) that the outbreak will cause me to be laid off or lose my job.
2. I fear (am worried) that the outbreak will lead to a worsening of my economic situation.
